# Understanding narwhal diving behaviour using Hidden Markov Models with dependent state distributions and long range dependence

**DOI:** 10.1371/journal.pcbi.1006425

**Published:** 2019-03-14

**Authors:** Manh Cuong Ngô, Mads Peter Heide-Jørgensen, Susanne Ditlevsen

**Affiliations:** 1 Greenland Institute of Natural Resources, Nuuk, Greenland; 2 Department of Mathematical Sciences, University of Copenhagen, Copenhagen, Denmark; 3 Greenland Institute of Natural Resources, c/o Greenland Representation, Copenhagen, Denmark; University of Pittsburgh, UNITED STATES

## Abstract

Diving behaviour of narwhals is still largely unknown. We use Hidden Markov models (HMMs) to describe the diving behaviour of a narwhal and fit the models to a three-dimensional response vector of maximum dive depth, duration of dives and post-dive surface time of 8,609 dives measured in East Greenland over 83 days, an extraordinarily long and rich data set. Narwhal diving patterns have not been analysed like this before, but in studies of other whale species, response variables have been assumed independent. We extend the existing models to allow for dependence between state distributions, and show that the dependence has an impact on the conclusions drawn about the diving behaviour. We try several HMMs with 2, 3 or 4 states, and with independent and dependent log-normal and gamma distributions, respectively, and different covariates to characterize dive patterns. In particular, diurnal patterns in diving behaviour is inferred, by using periodic B-splines with boundary knots in 0 and 24 hours.

## Author summary

Narwhals live in pristine environments. However, the increase in average temperatures in the Arctic and the concomitant loss of summer sea ice, as well as increased human activities, such as ship traffic and mineral exploration leading to increased noise pollution, are changing the environment, and therefore probably also the behavior and well-being of the narwhal. Here, we use probabilistic models to unravel the diving and feeding behavior of a male narwhal, tagged in East Greenland in 2013, and followed for more than two months. The goal is to gain knowledge of the whales’ normal behavior, to be able to later detect possible changes in behavior due to climatic changes and human influences. We find that the narwhal uses around two thirds of its time searching for food, it typically feeds during deep dives (more than 350*m*), and it can have extended periods, up to 3 days, without feeding activity.

## Introduction

The narwhal (*Monodon monoceros*) primarily inhabit cold waters of the Atlantic sector of the Arctic, with the largest abundances found in East and West Greenland and in the Canadian High Arctic [1]. The narwhal is one of the deepest diving cetaceans with the maximum exceeding 1800*m* [2], and it comes third only to Cuvier’s beaked whale *(Ziphius cavirostris)* (2992*m*) [3] and sperm whale *(Physeter macrocephalus)* (2035*m*) [4]. Narwhals dive to forage, and their diet consists of few prey species including Greenland halibut (*Reinhardtius hippoglossoides*), polar cod (*Boreogadus saida*), capelin (*Ammodytes villosus*) and squids (*Gonatus sp*.) [5, 6]. Narwhals depend on acoustics for sensing their environment, navigating and capturing prey at depth [7]. Anthropogenic factors like underwater noise are a concern for a species that, with decreasing sea ice coverage, is increasingly exposed to underwater noise from shipping and seismic exploration [8]. It is therefore important to understand and quantitatively describe the diving activities of narwhals, by robust statistical methods, to ensure the long-term conservation of one of the most specialized species in the North Atlantic.

The first step is to understand the diving patterns of narwhals under natural conditions, which we address in this study. Diving behaviour is however cryptic since it includes both physiological constraints, energetic demands and habitat and environmental regimes. Modelling of the observed diving behaviour is one way of gaining insight to the overall diving patterns, and changes in model parameters is a way to compare and estimate quantitatively changes in diving behavior or differences between individuals.

We apply multivariate Hidden Markov Models (HMMs) with covariates [9], to describe the diving dynamics in the vertical dimension of an individual narwhal. These types of models for similar diving data of Blainville’s beaked whales (*Mesoplodon densirostris*) were first introduced in [10]. A HMM assumes an underlying unobserved process, which governs the dynamics of the observed variables. The assumption is that the observed behaviour in a dive will depend on the present state, and introduces autocorrelation in the model [9]. These HMMs have been used for modelling animal movement by taking into account the correlation over time between different movement patterns, mainly in two horizontal dimensions (see, e.g., [11–13]), and recently, in one vertical dimension [10, 14], possibly including further information on vertical movements. In this study, we use vertical depth data, and the three response variables are the maximum depth reached in a dive, the duration of a dive, and the post-dive surface time before initiating a new dive.

In all previous studies, *contemporaneous conditional independence* was assumed, meaning that the state dependent processes are independent given the underlying state. This is a strong and often also an unrealistic assumption, since deeper dives will typically take longer. Even when conditioning the dive to be either shallow, medium or deep, a positive correlation is still expected, beyond the correlation implied by the hidden states. DeRuiter et al. [14] argued for the assumption of conditional independence because unless a multivariate normal distribution can be assumed, there is usually no simple candidate multivariate distribution to specify the correlation structure. This is partly due to some of their response variables being discrete. In this study, we will relax the assumption of conditional independence, taking advantage of the continuity of the response variables. They are all restricted to be positive and with right skewed distributions. Previous studies have therefore used conditionally independent gamma distributions for these variables. Here, we will assume dependent log-normal distributions, such that their log-transforms follow a multivariate normal distribution. We also do the analysis with the standard choice of the gamma distributions with both dependence and independence, as well as the independent log-normal distributions, and compare the results.

Covariates were included in [10, 13, 14], appearing in the transition probabilities between hidden states, whereas no covariates were included in [15]. Here we include covariates in all elements of the transition matrix, trying out different covariate process models and select the optimal model by the Akaike Information Criterion (AIC). We consider two covariates related to the recent deep dives performed by the narwhal. Dives can reach > 1800*m*, and deeper dives are assumed to be related to feeding [2]. We define a *deep dive* as a dive to a depth of at least 350*m*. One covariate is the time passed since the last deep dive, which was also used in [10]. The hypothesis is that the longer the time passed since last deep dive, the higher the narwhal’s propensity for initiating a deep dive will be. Another covariate counts the number of consecutive deep dives that the narwhal has performed. The hypothesis is that the more dives in a row and more time spent at great depths, the higher the narwhal’s propensity for changing diving pattern to shallower depth or near-surface travelling. By introducing such history dependent covariates, the model allows a longer dependence structure than the one implied by the Markov property. These models with dependencies between observables caused by the underlying state, as well as including feedback from the observed process, were introduced in [10] to model Blainville’s beaked whale. The last covariate is time of day at initiation of the dive, modelled by a periodic B-spline with boundary knots in 0 and 24 hours. Diurnal effects on marine mammal diving patterns are difficult to estimate in this type of models because the time series are typically too short. Here, we analyse a data set of a tagged narwhal that is extraordinarily long, nearly three months, making this inference possible. Normally, such time series are on the order of hours or days. However, we only have data from a single whale, and results might not generalize.

## Materials and methods

### Ethics statement

Permission for capturing, handling, and tagging of narwhals was provided by the Government of Greenland (Case ID 2010–035453, document number 429 926).

### Data

We analyse the time series of depth measurements of a mature male narwhal (420 cm, estimated mass 950 kg) tagged in East Greenland from August 13th until November 6th 2013. The tag (a satellite linked time depth recorder, the Mk10 time-depth recorder from Wildlife Computers, Redmond, WA, USA) was attached to the whale and retrieved one year later with 1994.83 hours of dive data (approximately 83 days and 2 hours), see [16]. In this time interval the narwhal performed 8,609 dives to depths of at least 20*m*. Depth was measured every second at a resolution of 0.5*m*, and preprocessed before analysis by summarizing in three variables within each dive to describe the behaviour: maximum depth (MD), dive duration (DT), and post-dive surface time (PD), as also used in [14]. A dive was scored every time the depth record went deeper than 20*m* (i.e., about four to six body lengths) to exclude brief shallow submersions between respirations, otherwise it is considered time spent at the surface, summarized in the variable PD. This threshold was chosen in order to avoid creating too many shallow dives near the surface, see [17]. We use a custom-written procedure in C++ combining with R [18] via Rcpp [19]. The dives are found by locating all zero depth measurements. If there is at least one depth measurement of at least 20*m* between two consecutive measurements of 0*m*, this is classified as a dive. Otherwise an interval between two 0*m* measurements is classified as part of the post-dive time after the last dive. For each identified dive, the largest depth measurement is defined as the maximum depth of the dive, and the dive duration is the time difference between the two 0*m* measurements. The surface and dive durations also enter in the model as part of the covariate counting the time since last deep dive.

In this study, the observed response variable, denoted by ***X***_*t*_, is three-dimensional, describing the diving behaviour related to each dive, where *t* indicates the dive number, *t* = 1, 2, …, *T*. The first response variable, *X*_1,*t*_, is MD reached in dive number *t*. The second response variable, *X*_2,*t*_, is DT of dive number *t*. The third response variable, *X*_3,*t*_, is PD after dive *t*. We assume that the diving behaviour depends on an underlying unobserved process, which we denote by *C*_*t*_, *t* = 1, 2, …, with a number *m* of unobserved behavioural states, *C*_*t*_ ∈ {1, …, *m*}, which govern the dynamics of the observed variables. The assumption is that the distributions of the observed MD, DT and PD of dive number *t* depend on the state.

### Hidden Markov Model

**Fig 1:**
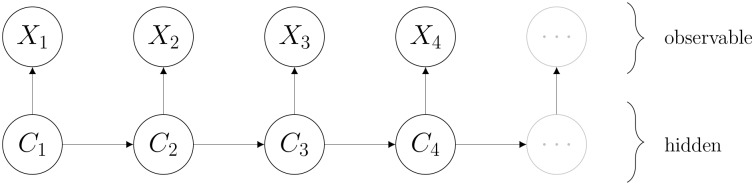
Hidden Markov Model. The hidden states *C*_*t*_ represent behavioural states that influence the distribution of the observed variables *X*_*t*_.

An *m*-dimensional hidden Markov model assumes that the distribution of the *p*-dimensional response vector ***X***_*t*_ depends on a hidden state *C*_*t*_, where {*C*_*t*_: *t* = 1, 2, …} is an unobserved underlying process satisfying the Markov property:

$$\begin{array}{c} P\left(C_{t}=j|C_{t-1}=i,\ldots ,C_{1}=l\right)=P\left(C_{t}=j|C_{t-1}=i\right), \end{array}$$

where *C*_*t*_ ∈ {1, …, *m*} for *t* = 2, 3, …. Denote the state transition probabilities at time *t* by *ω*_*ij*_(*t*), *i*, *j* = 1, …, *m*, where *ω*_*ij*_(*t*) = *P*(*C*_*t*+1_ = *j*∣*C*_*t*_ = *i*). The transition probability matrix Ω(*t*) is then

$$\begin{array}{c} \Omega \left(t\right)=[\begin{array}{ccc} \omega_{11}\left(t\right) & \cdots & \omega_{1m}\left(t\right)\\ \vdots & \ddots & \vdots \\ \omega_{m1}\left(t\right) & \cdots & \omega_{mm}\left(t\right) \end{array}] \end{array}$$ (1)

where *ω*_*ij*_(*t*) ≥ 0 and $\sum_{j=1}^{m}\omega_{ij}\left(t\right)=1$. Here, we let *ω*_*ij*_(*t*) depend on *t* to allow time varying covariates to affect the transition probabilities, see Section Covariates. The distribution of ***X***_*t*_ is conditionally independent of everything else given *C*_*t*_:

$$\begin{array}{c} f\left(X_{t}|X_{t-1},\ldots ,X_{1},C_{t},C_{t-1},\ldots ,C_{1}\right)=f\left(X_{t}|C_{t}\right),\;t=1,2,\ldots \end{array}$$ (2)

where *f* denotes a probability density function, i.e., the distribution of ***X***_*t*_ depends only on the current state *C*_*t*_ and not on previous states or observations. The model is illustrated in Fig 1.

### State dependent distributions

The state-dependent distributions are the probability density functions of ***X***_*t*_ associated with state *i*. Under the *contemporaneous conditional independence* assumption, the *p* different components of the response vector ***X***_*t*_ are assumed independent given the hidden state, and the probability density can be decomposed as

$$\begin{array}{c} f(X_{t}|C_{t}=i)=f_{i}(X_{t})=\prod_{k=1}^{p}f_{i,k}(X_{k,t}), \end{array}$$ (3)

where *X*_*k*,*t*_ is the *k*th observed component of ***X***_*t*_. Here we have *p* = 3, the components being MD, DT and PD. Thus, ***X***_*t*_ = (*X*_*MD*,*t*_, *X*_*DT*,*t*_, *X*_*PD*,*t*_)^*T*^, where ^*T*^ denotes transposition. *Contemporaneous conditional independence* implies that the state dependent processes *X*_*MD*,*t*_, *X*_*DT*,*t*_ and *X*_*PD*,*t*_ are independent given the underlying state *C*_*t*_. This assumption has been used in [14] and [15] because in general, there is no simple way to address the correlation between variables within states, and the dependence induced by the Markov chain is often sufficient to fit the data. However, in this paper, we will relax this assumption, and let *f*_*i*_ be a joint distribution function, allowing for dependent coordinates, which for our data turn out to improve the fit considerably.

All three response variables are positive right-skewed variables, so natural candidates for *f*_*i*,*k*_ are gamma distributions, as used in [14] and [15], or log-normal distributions, i.e., the logarithm of the response variables follow a 3-dimensional normal distribution. Here, we will try four different distributions. The first candidate is independent gamma distributions, to compare with the usual approach. The gamma distribution is parametrized by shape parameter *μ* and scale parameter *σ*, with mean *μσ* and variance *μσ*^2^, and the state dependent probability density functions are given by

$$\begin{array}{c} f_{i}\left(X_{t}\right)=\;\prod_{k\in \{MD,DT,PT\}}\;f_{i,k}(X_{k,t})=\;\prod_{k\in \{MD,DT,PT\}}\;\Gamma {\left(\mu_{i}^{k}\right)}^{-1}{\left(\sigma_{i}^{k}\right)}^{-\mu_{i}^{k}}X_{k,t}^{\mu_{i}^{k}-1}e^{-\frac{X_{k,t}}{\sigma_{i}^{k}}}, \end{array}$$ (4)

for *i* = 1, …, *m*.

We will also assume dependent gamma distributions [20] and both independent and correlated log-normal distributions, such that log ***X***_*t*_ is multivariate normal, where log ***X***_*t*_ = (log *X*_*MD*,*t*_, log *X*_*DT*,*t*_, log *X*_*PT*,*t*_)^*T*^, taking advantage of the computational convenience of the normal distribution. The log-normal distribution is parametrized by log-mean *μ* and log-variance *σ*^2^. Thus, given *C*_*t*_ = *i* and *k*, the mean and variance of log ***X***_*k*,*t*_ is $\mu_{i}^{k}$ and ${\left(\sigma_{i}^{k}\right)}^{2}$, and the mean and variance of ***X***_*k*,*t*_ is $\text{exp}(\mu_{i}^{k}+{\left(\sigma_{i}^{k}\right)}^{2}/2)$ and $\left(\text{exp}\left({\left(\sigma_{i}^{k}\right)}^{2}\right)-1\right)\text{exp}\left(2\mu_{i}^{k}+{\left(\sigma_{i}^{k}\right)}^{2}\right)$. The log-correlation between responses *k*_1_ and *k*_2_, for *k*_1_, *k*_2_ ∈ {*MD*, *DT*, *PT*} is denoted by $\rho_{i}^{k_{1},k_{2}}$. The correlation between components *k*_1_ and *k*_2_ is $\left(\text{exp}\left(\rho_{i}\sigma_{i}^{k_{1}}\sigma_{i}^{k_{2}}\right)-1\right)/\sqrt{\left(\text{exp}\left({\left(\sigma_{i}^{k_{1}}\right)}^{2}\right)-1\right)\left(\text{exp}\left({\left(\sigma_{i}^{k_{2}}\right)}^{2}\right)-1\right)}$, where ${\left(\sigma_{i}^{k_{1}}\right)}^{2}$ and ${\left(\sigma_{i}^{k_{2}}\right)}^{2}$ are the log-variances of *k*_1_ and *k*_2_, respectively. The correlation is approximately equal to the log-correlation $\rho_{i}^{k_{1},k_{2}}$ when ${\left(\sigma_{i}^{k_{1}}\right)}^{2}$ and ${\left(\sigma_{i}^{k_{2}}\right)}^{2}$ are small. Thus, the state dependent probability density functions are given by

$$\begin{array}{c} f_{i}\left(X_{t}\right)=\frac{1}{{\left(2\pi \right)}^{3/2}\sqrt{|\Sigma_{i}|}\cdot \prod_{k\in \{MD,DT,PT\}}\text{log}X_{k,t}}\text{exp}(-\frac{1}{2}{\left(\text{log}X_{t}-\mu_{i}\right)}^{⊤}\Sigma_{i}^{-1}\left(\text{log}X_{t}-\mu_{i}\right)), \end{array}$$ (5)

where |⋅| denotes the determinant of a matrix, $\mu_{i}={\left(\mu_{i}^{MD},\mu_{i}^{DT},\mu_{i}^{PD}\right)}^{T}$,

$$\begin{array}{c} \Sigma_{i}=[\begin{array}{ccc} {\left(\sigma_{i}^{MD}\right)}^{2} & \rho_{i}^{MD,DT}\sigma_{i}^{MD}\sigma_{i}^{DT} & \rho_{i}^{MD,PD}\sigma_{i}^{MD}\sigma_{i}^{PD}\\ \rho_{i}^{MD,DT}\sigma_{i}^{MD}\sigma_{i}^{DT} & {\left(\sigma_{i}^{DT}\right)}^{2} & \rho_{i}^{DT,PD}\sigma_{i}^{DT}\sigma_{i}^{PD}\\ \rho_{i}^{MD,PD}\sigma_{i}^{MD}\sigma_{i}^{PD} & \rho_{i}^{DT,PD}\sigma_{i}^{DT}\sigma_{i}^{PD} & {\left(\sigma_{i}^{PD}\right)}^{2} \end{array}] \end{array}$$

and $\rho_{i}^{k_{1},k_{2}}=0$ in the independent case.

### Covariates

**Fig 2:**
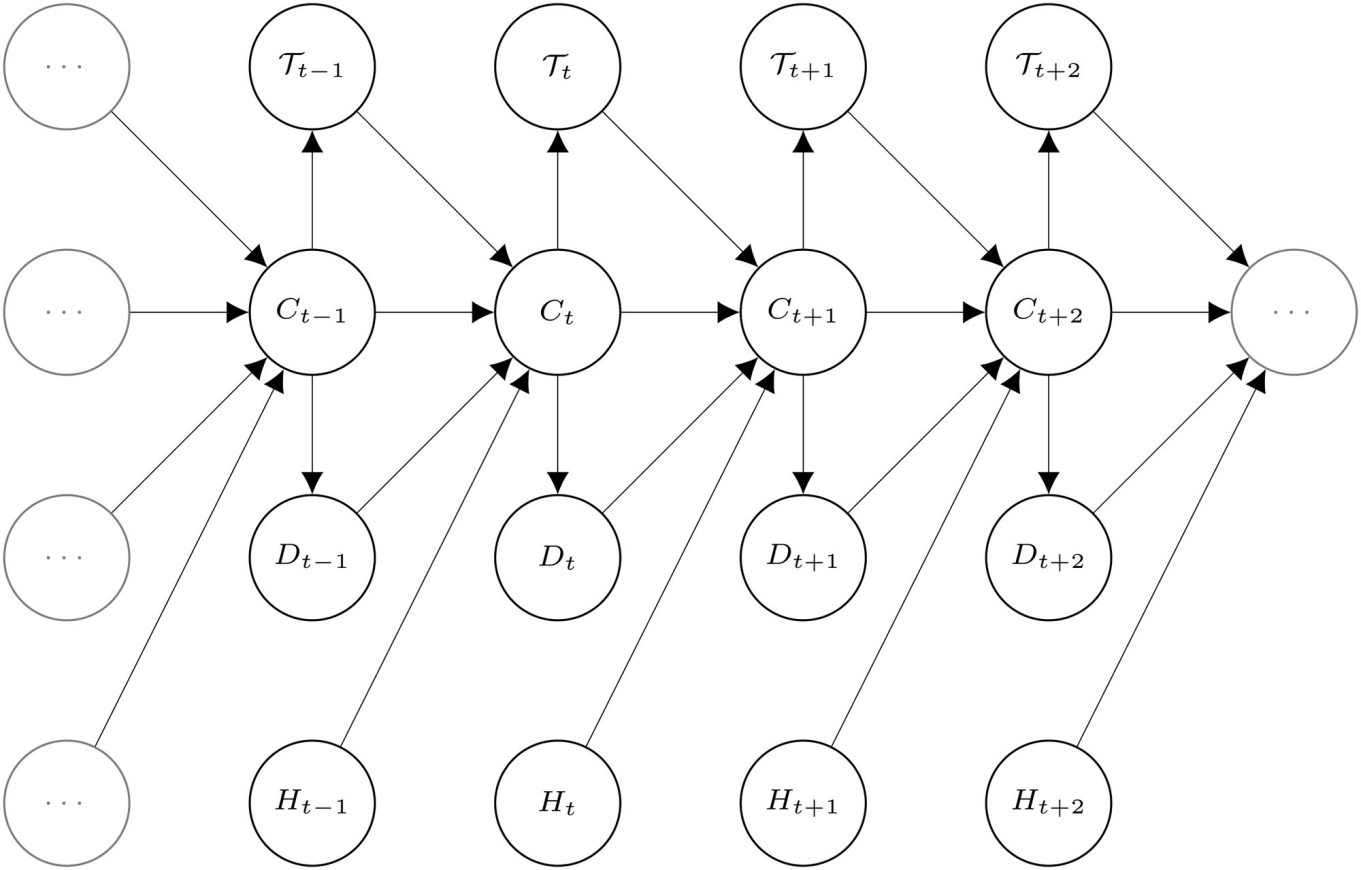
Hidden Markov Model with feedback processes. The transition probabilities between hidden states *C*_*t*_ depends on the observed covariate processes $T_{t}$, *D*_*t*_ and *H*_*t*_.

**Fig 3:**
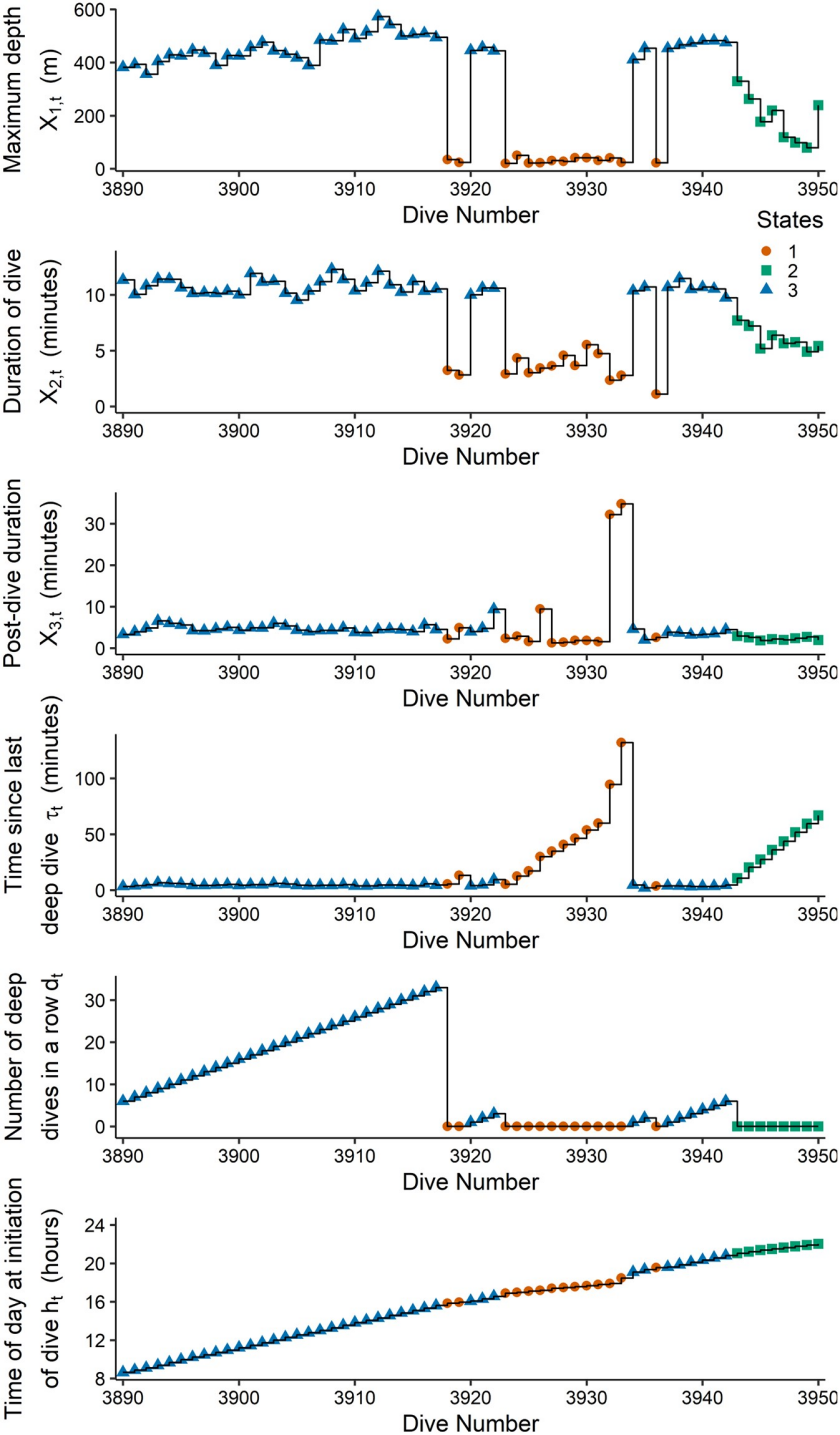
Response variables and covariate processes. Time series plot of maximum depth (MD), duration of dive (DT), and post-dive duration (PD) from dive number 3890 to 3950 and the covariate processes counting the time since last deep dive (*τ*_*t*_), number of deep dives in a row (*d*_*t*_), and the hour at initiation of dive (*h*_*t*_). The symbols indicate the decoded hidden states from a model fitted to a dependent log-normal distribution (Model 1).

To allow for a longer memory in the model beyond the autocorrelation induced by the hidden process, we incorporate feedback mechanisms by letting the state transition probabilities depend on the history. We consider two covariates related to the recent deep dives performed by the narwhal. One covariate is the continuous variable *τ*_*t*_, defined as time passed since the last deep dive before dive number *t*, where a *deep dive* is defined as a dive to a depth of at least 350*m*. Maximum depths are bimodal, and the value is chosen as a lower threshold of the deeper dives. Note that this definition is only used to define the covariates, and is not related to the decoding of states. The other covariate is the discrete variable *d*_*t*_ taking non-negative integer values, counting the number of consecutive deep dives that the narwhal has performed before dive number *t*. Thus, covariate *τ*_*t*_ measures physical time since last deep dive, whereas covariate *d*_*t*_ counts number of deep dives in a row, independently of time passed. Finally, we consider the covariate of the hour of the day at which the dive is initiated. More specifically, we define the covariate processes $T_{t}$, the time since the last deep dive, *D*_*t*_, the number of consecutive deep dives up to dive number *t*, and *H*_*t*_, the hour of initiation of dive *t*, and denote the measured covariates by *τ*_*t*_, *d*_*t*_ and *h*_*t*_. Thus, the short term memory is modelled by the hidden states, and the long term memory is modelled by modulation of the transition probabilities as a function of past dynamics. The model is illustrated in Fig 2. Fig 3 illustrates the response variables and the three covariates for 60 consecutive dives.

The covariates enter the transition probabilities *ω*_*ij*_(*t*) = *ω*_*ij*_(*η*_*ij*_(*t*)) in Eq (1) through a *predictor*, *η*_*ij*_(*t*), see Eq (7) below. We consider several models. If there are no covariates for a given predictor, then *η*_*ij*_(*t*) = *η*_*ij*_ does not depend on *t*. In S1 Table in the Supporting Information, all the covariate models that were fitted are listed, where *α*_*ij*_, *β*_*ij*_, *γ*_*ij*_, *δ*_*ij*_, *θ*_*ij*_ and *ζ*_*ij*_ are real parameters. Covariates *d*_*t*_ and *τ*_*t*_ were incorporated as natural cubic splines with three degrees of freedom. The effect of time of day is modelled by a periodic B-spline with three degrees of freedom, with boundary knots in 0 and 24 hours.

### The likelihood function and optimization

The likelihood *L*_*T*_ of *x*_1_, *x*_2_, …, *x*_*T*_, where *x*_*t*_ is the observation of ***X***_*t*_, assumed to be generated by an *m*-state HMM, can in general be computed recursively in only *O*(*Tm*^2^) operations by the forward algorithm [9]. The likelihood is expressed as

$$\begin{array}{c} L_{T}=\delta P\left(x_{1}\right)\Omega \left(\tau_{1},d_{1},h_{1}\right)P\left(x_{2}\right)\cdots \Omega \left(\tau_{T-1},d_{T-1},h_{T-1}\right)P\left(x_{T}\right)1, \end{array}$$ (6)

where $P\left(x_{t}\right)=\text{diag}\left(f_{1}\left(x_{t}\right),\ldots ,f_{m}\left(x_{t}\right)\right)$ is a diagonal matrix with diagonal elements *f*_*i*_(*x*_*t*_) given in Eq (4) when the gamma distribution is used, or Eq (5) when the log-normal distribution is used, Ω is given by Eq (1) and $1\in R^{m}$ is a column vector of ones. The initial state distribution is denoted by *δ*, which is an *m*-dimensional row vector; *δ*_*i*_ = *P*(*C*_1_ = *i*). For *δ*, we choose the uniform distribution, *δ*_*i*_ = 1/*m*. Alternatively, it can be estimated, but there is no need for this extra computational effort, since our dataset is large and the influence of *δ* will be negligible. To test this hypothesis, we repeated the optimization with the optimized parameters as initial condition, only changing the distribution of *δ* to the decoded distribution at time 1. This did not change the estimates. Furthermore, *δ* has no particular biological relevance.

The transition parameters in Eq (1) are constrained to be between 0 and 1 with row sums equal to 1, and thus, even if there are *m*^2^ entries, there are only *m* ⋅ (*m* − 1) free parameters. To obtain an unconstrained optimization problem, we reparametrise to working parameters, as also done in [13–15], see also [9], by defining

$$\begin{array}{c} \omega_{ij}\left(t\right)=\frac{\text{exp}(\eta_{ij}\left(t\right))}{\sum_{j=1}^{m}\text{exp}\left(\eta_{ij}\left(t\right)\right)} \end{array}$$ (7)

where *η*_*ij*_(*t*) is the predictor for dive *t* for 1 ≤ *i*, *j* ≤ 3, *i* ≠ *j*, and *η*_*ii*_ = 0 for *i* = 1, 2, 3. This assures positive entries and that rows sum to 1.

We used the direct numerical Newton-Raphson algorithm nlm (optim in case nlm failed) in R [18] to estimate the parameters of the model by maximizing the log-likelihood, $L_{T}≔\text{log}\;L_{T}$, where *L*_*T*_ is given in Eq (6). The procedure ns from the package splines (version 3.5.0) was used to calculate the natural cubic splines. The procedure pbs from the package pbs (version 1.1) was used to calculate the periodic splines.

Using a combination of R and Rcpp [19] for calculating the log-likelihood function $L_{T}$ improved the runtime considerably. To mitigate the problem of local maxima, we ran the optimization algorithm up to a thousand times with different starting values for the parameters. The starting values were chosen as follows. For the parameters of the state-dependent distributions, an independent mixture model was fitted to the response distributions, and the estimated parameters were used as initial conditions. In the correlated models, the correlation parameter between MD and DT was initiated at the empirical correlation in the data set. The parameters of the covariates were varied in a regular grid together with the jittering procedure used in [14], such that they looped through 0 to ±5 in steps of 1 for *α*_*ij*_, *β*_*ij*_ and *γ*_*ij*_. The final result was chosen as the one giving the maximum log-likelihood.

The best model fit was evaluated by AIC. Once the optimal model was selected and parameters of the model were estimated, it was of interest to decode the most likely state sequence $c_{1}^{*},\ldots ,c_{T}^{*}$. The Viterbi algorithm [9, 21] was used to estimate the hidden states given the observed depths and durations:

$$\begin{array}{c} (c_{1}^{*},\ldots ,c_{T}^{*})=\underset{(c_{1},\ldots ,c_{T})\in \left\{1,\ldots ,m\right\}}{\text{argmax}}\text{Pr}(C_{1}=c_{1},\ldots ,C_{T}=c_{T}|x_{0},\ldots ,x_{T}). \end{array}$$

## Results

**Fig 4:**
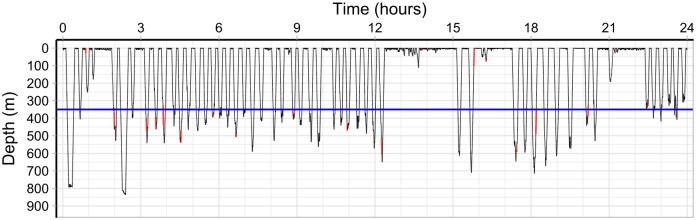
Diving data. Representative part of the narwhal diving data, covering 24 hours of dives on August 15th 2013. The red parts are where a lower temperature in the stomach has been registered, indicating that the narwhal has swallowed a prey. The blue line indicates a depth of 350*m*, the threshold for a *deep dive* used in the definition of the covariates.

The data set covers 1,995 hours (∼ 83 days) with *T* = 8, 609 dives, and is extraordinarily long, and thus provides a unique opportunity to obtain detailed information on diving behaviour. An example of the data is shown in Fig 4. Such data are usually only on the order of a couple of days or less, for example, the time series of short-finned pilot whales *(Globicephala macrorhynchus)* analysed in [15] cover up to 18 hours and 64 dives, whereas the time series of blue whales *(Balaenoptera musculus)* analysed in [14] cover up to 6 hours and 67 dives, and Langrock et al. [12] analyses 79 hours of a single Blainville’s beaked whale. Detailed diving data of narwhals are available for up to 33 hours [6] or up to one week [7]. However, here we only have data from a single narwhal limiting the generalizability of the analysis.

The first week of tagging, the narwhal also had the temperature of the stomach measured, see [22]. A temperature drop indicates that a prey has entered the stomach. The red parts in Fig 4 indicate temperature drops. These typically happen during deep dives, and support the assumption that deep dives are related to foraging. This is also supported by the findings in [7], where buzzes, related to foraging, are typically produced when the whales are at 200–600*m*.

**Fig 5:**
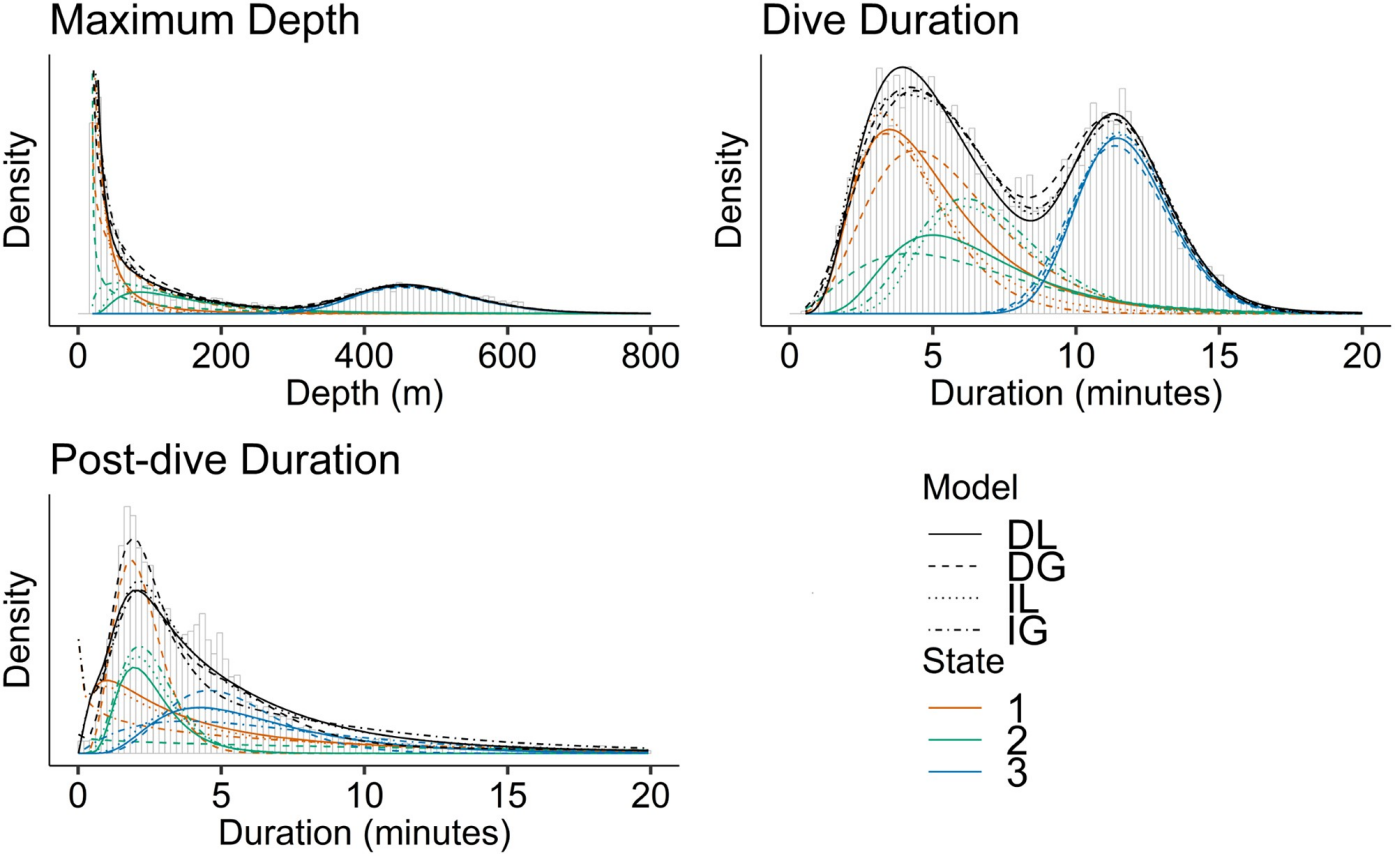
Model fit. Histograms of response variables MD, DT and PD. The fit of Model 1 is indicated with black curves, for dependent lognormal (DL), independent lognormal (IL), dependent gamma (DG) and independent gamma (IG). The distribution of the fitted states are indicated with colours as given in the legend. State 1 corresponds to near surface, state 2 medium depths, and state 3 large depths.

The variable MD takes values between 20 and 910.5*m*, DT takes values between 33 seconds and 28 minutes, and PD takes values between 1 second and 209.7 minutes. Fig 5 shows histograms of the three response variables. Maximum depths are bimodal and typically either less than 200*m* or between 400 and 600*m*. This was used to select the threshold of 350*m* to define a deep dive. The value is chosen as a lower threshold of the deeper dives. We furthermore tried different values between 250 and 450*m* in steps of 50*m*. The results only changed very little within this range, and thus, the analysis is robust to the choice of threshold.

To choose the number of states *m*, we optimized models with each of the four state distributions for *m* = 2, 3 and 4 states, including all covariates. Since the gamma model is computationally very expensive, and furthermore does not provide a better fit, we only ran the gamma models for *m* = 2 and 3. Typical runtimes are given in Table 1. The runtimes vary over many orders of magnitudes. For all state distributions, the 4-state model takes on the order of hours to run, which makes it infeasible, since for each covariate model, many repetitions from different starting conditions have to be run, and the number of needed repetitions explode as the number of parameters increase. Moreover, the 4-state model did not improve qq-plots, as shown later. The 3-state correlated gamma model is also very slow and not feasible to use if many covariate models should be explored. In general, the log-normal model is much faster than the gamma model, and the computational cost of including dependence is small. It is not obvious if a 2 or a 3-state model should be chosen. However, the runtimes for the 3-state model are acceptable, and based on both qq-plots and AIC values presented below, the 3-state HMM is preferred. Thus, similar to the blue whales data analysed in [14], our narwhal data suggest three distinct states. Pohle et al. [23] recommended against using more than four states in biological modelling like this, in order to avoid the complexity of the correspondence between states of the model and the biological phenomenon. DeRuiter et al. [14] suggested three states for their data, even if a formal model selection procedure would point to a more complex model, because models with more underlying states might obscure patterns in the data and provide less insight in the underlying biological process, even if they might perform better in terms of forecasting. Biological knowledge should guide the choice of number of states. They also argue that model misspecifications, such as too inflexible state dependent distributions, variations over time, missing covariate information or outliers might cause model selection criteria to favour models with more complex structures than warranted. Therefore, we choose the 3-state HMM. The algorithm allocates labels arbitrarily, so to compare across models we relabelled the states, such that state 1 represents the shortest and shallowest dives, which we interpret as near-surface travelling, social activities and resting, state 2 represents medium long and deep dives, which we identify with a feeding state for prey located at medium depths, and state 3 represents the deepest and longest dives, which we identify with a feeding state for prey located at deep depths.

**Table 1:** Complexity of models. Runtimes and number of variables for different state distributions and for 2, 3 and 4 states for covariate model 1. Runtimes are on Intel Xeon E5-2697v2 @ 2.7 GHz.

	No. of variables	Range of runtime	Average of runtime
Correlated log-normal			
2-state	28	0.9–3 (min)	1.9 (min)
3-state	63	2.25–15 (min)	7.3 (min)
4-state	112	1–2.4 (hrs)	1.6 (hrs)
Correlated gamma			
2-state	28	1.14–9.30 (min)	5.65 (min)
3-state	63	17.25–86.81 (min)	54.88 (min)
Independent log-normal			
2-state	26	0.11–3.66 (min)	1.28 (min)
3-state	60	3–12.56 (min)	5.7 (min)
4-state	108	1–3 (hrs)	1.88 (hrs)
Independent Gamma			
2-state	26	1.58–3.57 (min)	2.43 (min)
3-state	60	11.81–26.35 (min)	15.53 (min)

The empirical correlations between response variables in the full data set are small for MD and PD (0.046), and for DT and PD (0.042), only the correlation between MD and DT is significant (0.86). If the data set is split into three subsets according to MD, namely for MD between 20 and 50 m, for MD between 50 and 350 m, and for MD above 350 m, these results still hold. All correlations involving PD in all groups are less than 0.11 in absolute values, whereas the correlations between MD and DT are 0.27, 0.58 and 0.41, respectively. We therefore only assumed dependence between MD and DT. This improved convergence and runtime. To check that this assumption is reasonable, covariate model 1 with 3 states was fitted to the fully correlated log-normal model, and all estimated correlations with PD were smaller than 0.14, except for state 2, where it was around 0.5. The other estimates did not change compared to a model with only correlation between MD and DT.

We tried a total of 14 covariate models, listed in S1 Table in the Supporting Information. Here, we only include the best model based on the AIC criteria (model 1), and 3 more models for illustration (Table 2).

**Table 2:** Different models for covariate effects on the transition probabilities between behavioural states. The predictors *η*_*ij*_ relate to the transition probabilities as given in Eq (7). The spline effects of hour are denoted by $H_{ij}^{t}=\sum_{k}\delta_{ij}^{\left(k\right)}h_{k}^{t}$, of *τ*_*t*_ by $T_{ij}^{t}=\sum_{k}\theta_{ij}^{\left(k\right)}s_{k}^{t}$, and of *d*_*t*_ by $D_{ij}^{t}=\sum_{k}\zeta_{ij}^{\left(k\right)}d_{k}^{t}$ for *k* = 1, 2, 3 and *i*, *j* = 1, 2, 3; *i* ≠ *j*. A list of all explored models can be found in S1 Table in the Supporting Information.

Predictors in the transition probabilities						
Model	*η*_12_(*t*)	*η*_13_(*t*)	*η*_21_(*t*)	*η*_23_(*t*)	*η*_31_(*t*)	*η*_32_(*t*)
1	$\alpha_{00}+T_{12}^{t}+H_{12}^{t}$	$\alpha_{01}+T_{13}^{t}+H_{13}^{t}$	$\beta_{00}+T_{21}^{t}+H_{21}^{t}$	$\beta_{01}+T_{23}^{t}+H_{23}^{t}$	$\gamma_{00}+D_{31}^{t}+H_{31}^{t}$	$\gamma_{01}+D_{32}^{t}+H_{32}^{t}$
2	$\alpha_{00}+H_{12}^{t}$	$\alpha_{01}+H_{13}^{t}$	$\beta_{00}+H_{21}^{t}$	$\beta_{01}+H_{23}^{t}$	$\gamma_{00}+H_{31}^{t}$	$\gamma_{01}+H_{32}^{t}$
3	$\alpha_{00}+T_{12}^{t}$	$\alpha_{01}+T_{13}^{t}$	$\beta_{00}+T_{21}^{t}$	$\beta_{01}+T_{23}^{t}$	$\gamma_{00}+D_{31}^{t}+H_{31}^{t}$	$\gamma_{01}+D_{32}^{t}+H_{32}^{t}$
4	$\alpha_{00}+T_{12}^{t}$	$\alpha_{01}+T_{13}^{t}$	$\beta_{00}+T_{21}^{t}$	$\beta_{01}+T_{23}^{t}$	$\gamma_{00}+D_{31}^{t}$	$\gamma_{01}+D_{32}^{t}$

Model 1 has diurnal effects on all transition probabilities, and nonlinear effects of *τ*_*t*_ and *d*_*t*_ on some of the transition probabilities. The covariate *d*_*t*_ counts number of deep dives in a row, and is therefore around 0 when not in state 3. This covariate therefore carries no information unless in state 3, and only enters in *η*_31_ and *η*_32_. Likewise, *τ*_*t*_ is expected to be around 0 when in state 3, and therefore only enters *η*_*ij*_ for *i* = 1 or 2. Model 2 only has diurnal effects. Model 3 has effects of the dive covariates, but only diurnal effects in state 3. Finally, model 4 has only dive effects and no diurnal effects.

Table 3 lists the model selection results from the optimization. We use AIC to select the best model, which is highlighted in bold. The correlated log-normal model is clearly preferred above the other models, with huge AIC differences. The dependent models are clearly preferred above the independent models, and the log-normal distribution is clearly preferred above the gamma distribution. Models with ΔAIC larger than 10 have essentially no support in the data compared to the best model [24]. Model 1 is the best among the tested models for all state distribution models, which balance accuracy and complexity of the model. The marginal fit of covariate model 1 is illustrated in Fig 5 for the four state distributions, where the black curves provide the overall distributions of the three response variables, as well as the distributions within each state. The fits look convincing for MD and DT, whereas the models capture the bimodality of PD less well. Note that the splitting into states 1 and 2 depends on the state distributions, whereas the distributions of state 3 are approximately the same for all state distributions. Thus, the classification of behavioral states will depend on the chosen state distribution mainly for small and medium dives.

**Table 3:** Model selection results. Differences in AIC values, ΔAIC = AIC—AIC_*min*_, between the different models with 3 hidden states, where AIC_*min*_ is the value of the model with the lowest AIC. The best fit is given by the minimum AIC. For all the tested state distributions, covariate model 1 was preferred, and for all covariate models, the dependent log-normal state distribution was preferred. Because the runtimes for the correlated gamma model are high, only Model 1 was fitted. The best model is highlighted in bold. *np*: number of parameters.

	Independent Gamma distribution		Independent Log-normal distribution		Correlated Gamma distribution		Correlated Log-normal distribution	
Model	*np*	ΔAIC	*np*	ΔAIC	*np*	ΔAIC	*np*	ΔAIC
**1**	60	5050.5	60	2309.1	63	1901.9	**63**	**0**
2	42	5386.9	42	2652.4	45	-	45	256.0
3	48	5096.5	48	2353.2	51	-	51	34.3
4	42	5194.4	42	2451.9	45	-	45	166.9

**Fig 6:**
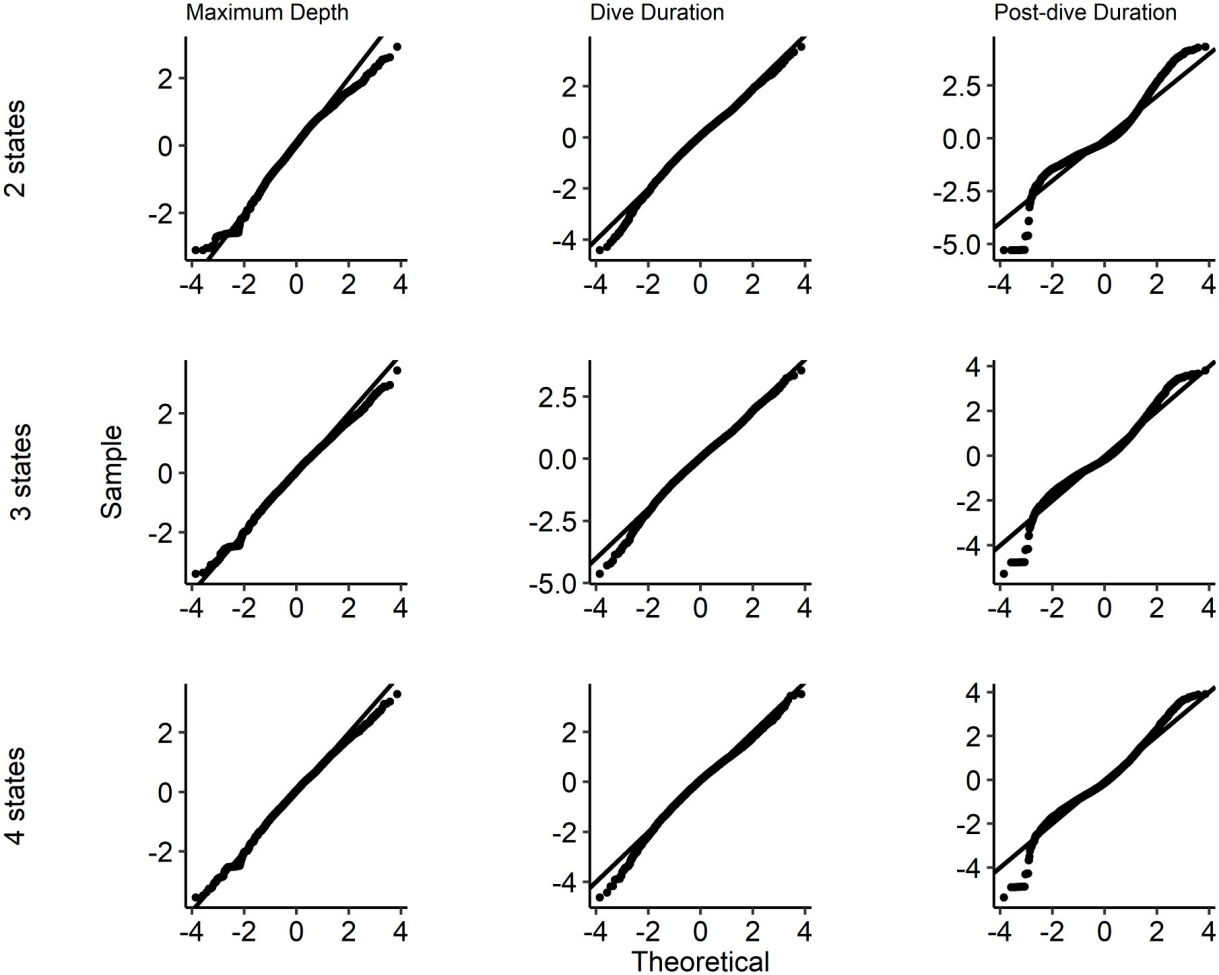
Quantile-quantile residual plots. QQ-plots of forecast pseudo-residuals from covariate model 1 with correlated log-normal state distribution.

To check the fit of the model beyond what is presented in Fig 5, we calculated the pseudo-residuals [9] and made qq-plots (Fig 6) for the correlated log-normal model with *m* = 2, 3 and 4 states. The other state distributions give similar qq-plots, and are therefore omitted. A slight improvement is observed when passing from 2 to 3 states, in particular for PD. The fit does not improve when passing from 3 to 4 states. The fit is acceptable for MD and DT, maybe except for a too small lower tail for the MD. This is probably due to the threshold of a depth of 20*m* in the definition of a dive. The PD is less well fitted, especially in the lower tail, which could also be partly due to the cut-off threshold of 20*m* in the definition of PD. It is acceptable for 3 and 4 states.

**Fig 7:**
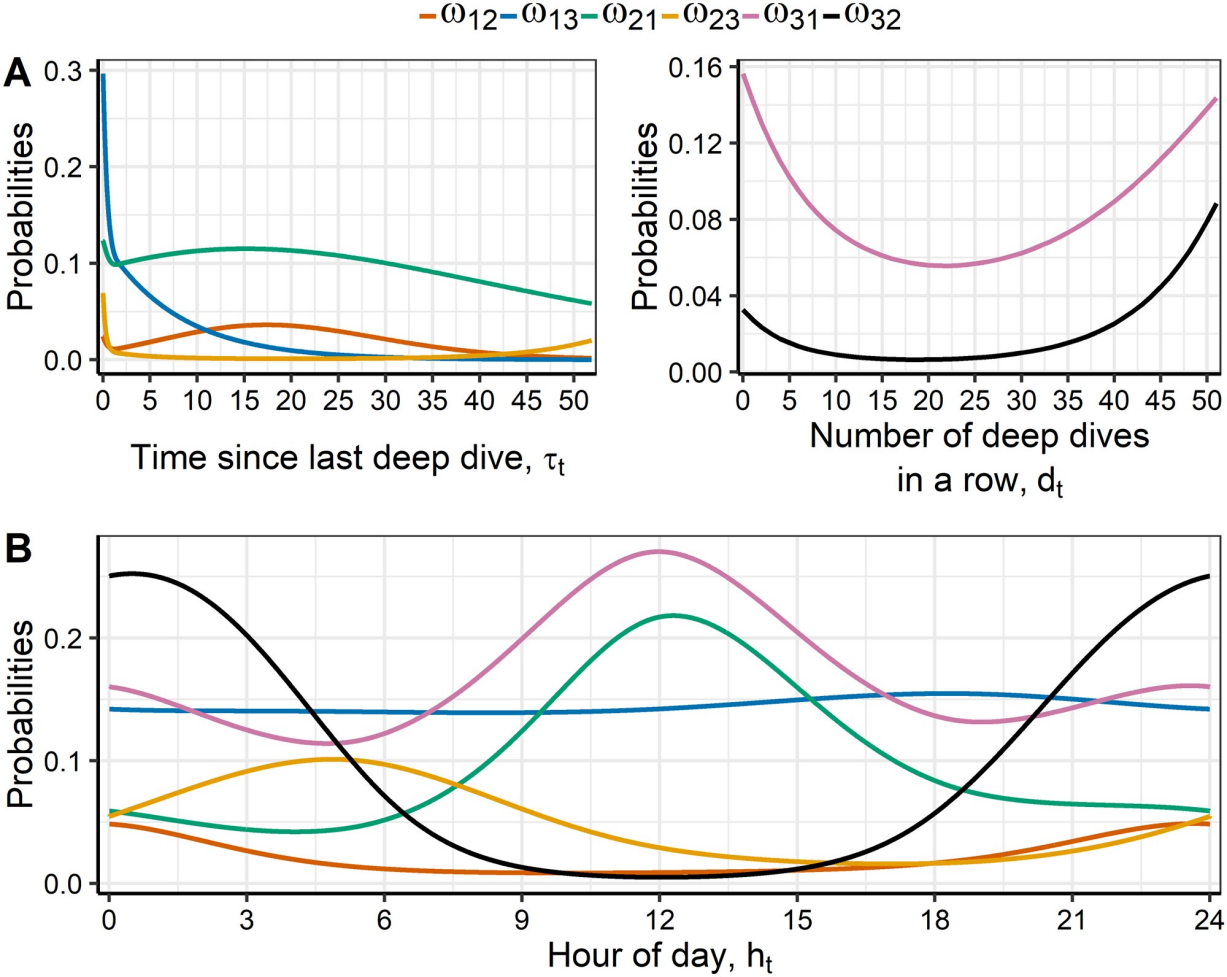
Covariate effects. A: Transition probabilities between behavioural states depending on covariates related to deep dives of correlated log-normal model 1, at approximately 12 pm. B: Transition probabilities depending on diurnal effects in model 1 with correlated log-normal state distributions, calculated for *τ*_*t*_ = 0.58 and *d*_*t*_ = 0 (the medians).

Fig 7 illustrates the estimated covariate effects for the optimal model, the correlated log-normal state distributions with covariate model 1. Parameter estimates and confidence intervals can be found in S2 and S3 Tables in the Supporting Information.

**Fig 8:**
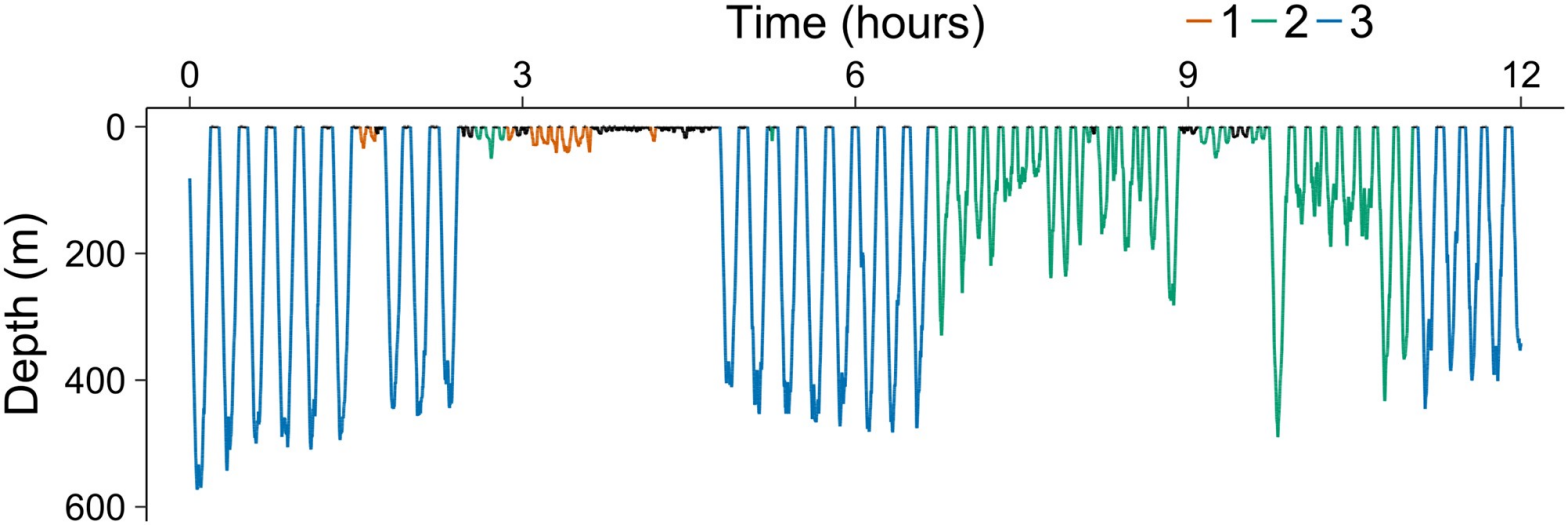
State decoding close-up. The estimated hidden state per dive for 12 hours of the data, starting on 22 September 2013 at 14:18:39.

The covariate *τ*_*t*_ indicates the time passed since last deep dive. We expect that *τ*_*t*_ has impacts on states 1 and 2, but not on state 3 (which is the case for the selected model). In the left panel of Fig 7A the effect of *τ*_*t*_ is illustrated. The transition probabilities do not seem to depend much on *τ*_*t*_, except for the probability of changing from state 1 to state 3. The probability is higher for small values of *τ*_*t*_, and decreasing fast towards 0 for larger values. This is not what was expected, but might reflect the following. When short time has passed since last deep dive, it was probably also a short time since the whale was in state 3. Thus, it reflects that the whale is still in an overall behavioral state 3, but just had a short break in state 1. This phenomenon can be seen in Fig 8 where the state decoding is shown for 12 representative hours. It is seen that after (at least) six dives in state 3, the whale changes to a few shallow dives for a short time, and then continues with another three dives in state 3. When a little longer time passes, the whale has effectively stopped diving deep, and the probability of a change to state 3 becomes smaller. Then, when long time has passed, we expect the transition probability to increase, which is not what is estimated. However, there are few large observations of *τ*_*t*_: 75% of the values are below 2.8 hours, and 90% are below 7.8 hours. Therefore, the estimates of covariate effects for large values are unreliable. The effect of *d*_*t*_ is illustrated in the right panel of Fig 7A. As expected, for values above 20 dives in a row, the probabilities to exit state 3 increase with increasing *d*_*t*_. However, the data is sparse for large values of *d*_*t*_ and estimates might not be trusted: more than half are 0, 75% are 2 or smaller, and 90% are 8 or lower. The probability of changing to state 1 is much higher than the probability of changing to state 2 after a period in state 3.

Fig 7B shows the diurnal effects on the transition probabilities. Changing from state 3 to 2 has highest probability around midnight, whereas changing from state 2 to 3 has highest probability around 6 am. Changing to state 1 has highest probability around noon. The transition probabilities from state 1 do not depend much on diurnal effects.

Table 4 lists the estimated means and standard deviations of the four state distributions. Means and standard deviations of maximum depth are estimated larger for both state 1 and state 2 with the correlated models compared to the independent models, whereas all models estimate mean and variances approximately the same for state 3. Thus, taking into account the dependence between the two state variables reveals more variable diving patterns (i.e., larger variance within states), unless the narwhal is doing deep dives in state 3, where the need for regular breathing do not allow the whale to make detours. In general, the distributions of the response variables within states change depending on the assumed state distributions, and whether correlation is accounted for or not. To understand the classification of behavioural states provided by the HMM, we also added the empirical measures from the data decomposed into three subsets according to maximum depth: state 1 defined as dives between 20 and 50*m*, state 2 defined as dives between 50 and 350*m*, and state 3 for dives of more than 350*m*. This shows that none of the HMMs classifies the dives only according to depth, since these empirical measures differ from all the estimated distributions. Thus, the HMMs might reveal more complex behavioural states than given by the diving depths.

**Table 4:** Summary measures of Model 1 with 3 states. Means and standard deviations based on correlated Log-normal, correlated Gamma, independent Log-normal and independent Gamma distribution. MD: Maximum Depth; DT: Diving Time; PD: Post-Dive duration. E: mean; SD: standard deviation; Corr_1_: Correlation between MD and and DT. Corr_2_: Correlation between MD and and PD. Corr_3_: Correlation between DT and and PD. The empirical distribution is the empirical measures in three subgroups of the data classified according to MD, state 1: MD between 20 and 50 m, state 2: MD between 50 and 350 m, state 3: MD above 350 m.

	State 1	State 2	State 3
**Correlated Log-normal distribution**			
E_*MD*_	51.04	174.19	479.29
SD_*MD*_	57.54	109.09	81.36
E_*DT*_	5.05	6.54	11.79
SD_*DT*_	2.61	2.52	1.65
E_*PD*_	7.56	2.58	6.93
SD_*PD*_	14.85	1.23	7.45
Corr_1_	0.56	0.81	0.46
**Correlated Gamma distribution**			
E_*MD*_	88.46	112.37	471.81
SD_*MD*_	78.60	153.96	83.03
E_*DT*_	5.50	5.95	11.60
SD_*DT*_	2.49	3.48	1.72
E_*PD*_	2.19	16.03	5.36
SD_*PD*_	0.87	20.43	2.29
Corr_1_	0.59	0.80	0.53
**Independent Log-normal distribution**			
E_*MD*_	42.68	150.29	477.37
SD_*MD*_	34.53	89.87	83.50
E_*DT*_	4.37	7.00	11.81
SD_*DT*_	2.11	2.07	1.70
E_*PD*_	7.64	2.66	7.18
SD_*PD*_	14.77	1.25	8.99
**Independent Gamma distribution**			
E_*MD*_	39.47	133.14	474.87
SD_*MD*_	25.86	87.27	85.80
E_*DT*_	4.10	6.85	11.77
SD_*DT*_	1.81	2.23	1.72
E_*PD*_	8.15	2.55	7.34
SD_*PD*_	15.13	1.12	9.65
**Empirical distribution**			
E_*MD*_	30.87	143.52	484.67
SD_*MD*_	8.45	86.16	77.14
E_*DT*_	4.25	6.73	11.83
SD_*DT*_	2.04	2.43	1.72
E_*PD*_	7.07	4.43	7.18
SD_*PD*_	13.99	8.45	9.44
Corr_1_	0.27	0.58	0.41
Corr_2_	-0.11	0.07	0.05
Corr_3_	-0.01	0.08	0.06

The Viterbi algorithm classifies each dive to one of the three hidden states. The classification depends on the model, but all models roughly group dives according to maximum depth. One goal of comparing models is to access if conclusions on diving behaviour expressed through the decoded classes of the dives differ between models. If they all classify the same, it does not matter which model we use, maybe except for the estimation of covariate effects. If the classification differ from model to model, it is important to choose the statistically best model, measured from AIC, qq-plots, runtimes and biological interpretability.

**Fig 9:**
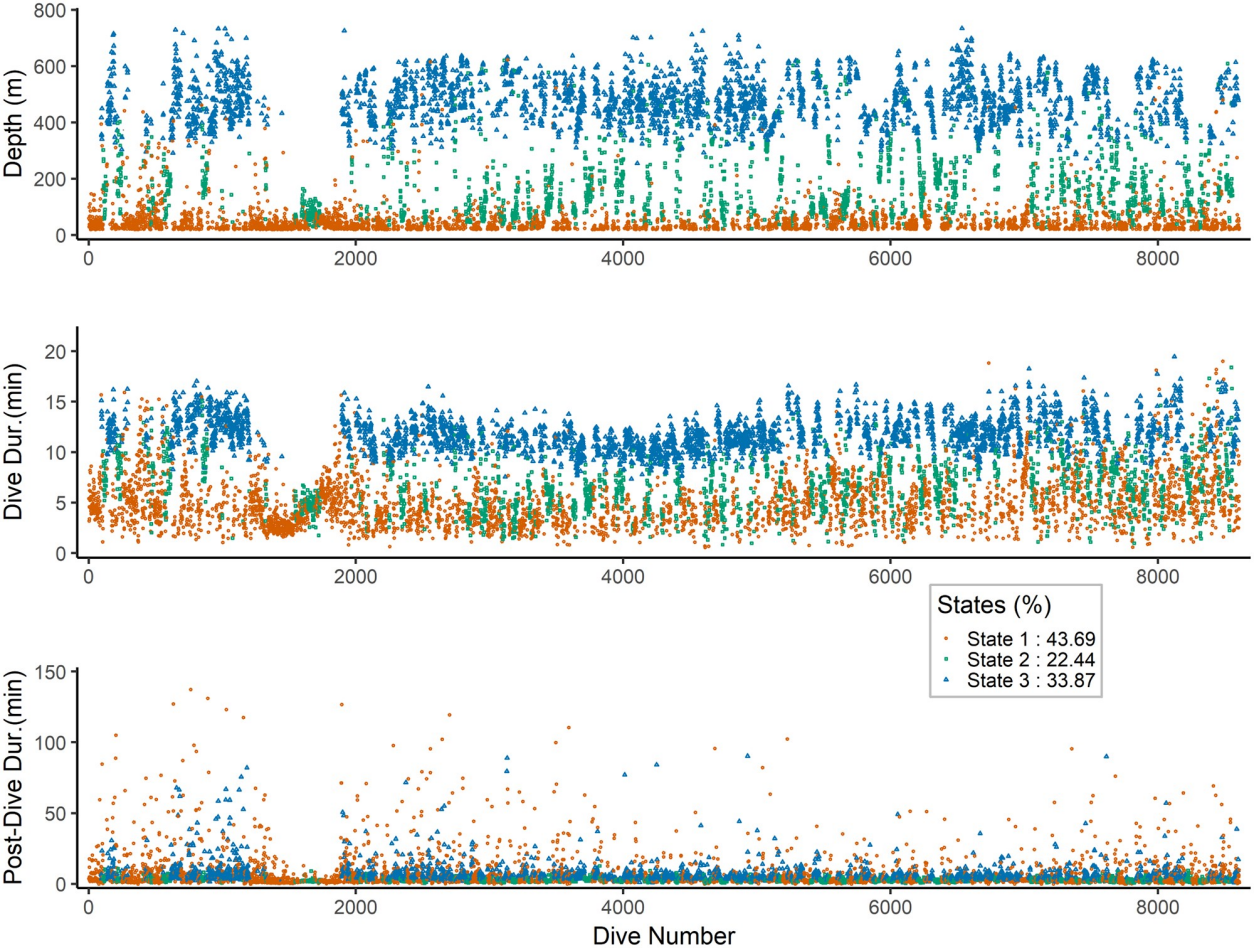
State decoding. The estimated hidden state per dive for each of the three observed variables under covariate model 1 and state distribution the correlated log-normal. The longest pause of no deep dives starts from the 1345th dive until the 1894th dive, and it lasts approximately 2 days and 17.5 hours.

Fig 9 shows the decoded hidden states for Model 1 with dependent log-normal state distribution. The correlated log-normal model estimates that the narwhal spends around 43.7% of its dives, corresponding to 28.8% of the time in State 1, which encompasses dives down to 793*m* of durations up to 28 minutes. This is a large value for the surface state, but it is only the extreme tail of the distribution, and is represented by a single dive. It reflects that the log-normal distribution has heavier tails than the gamma distribution, and that the behavioural states are more complex than what can be explained only by maximum depth. Of the time spent in state 1, only 15.9% of the time is spent diving, the rest of the time the whale is at the surface. The narwhal spends around 22.4% of its dives, corresponding to 19.2% of the time, in medium depths of between 22.5*m* and 836*m* and durations between 0.8 and 21.3 minutes. Also here, a few deep dives are decoded as belonging to state 2. Of the time spent in state 2, 10.6% of the time is spent diving, the rest of the time the whale is at the surface. Finally, 33.9% of dives, corresponding to 52.1% of the time, are spent in state 3 at depths between 243*m* and 910.5*m* and durations between 7.2 and 19.5 minutes. Of the time spent in state 3, 28.9% of the time is spent diving, the rest of the time the whale is at the surface. Fig 8 illustrates a close-up of the decoding of dives for an example period of 12 hours. The correlated model thus decodes a few of the deep dives as pertaining to states 1 and 2, probably because of these dives taking longer time than the deep dives decoded as state 3.

Apparently the whale could stay in state 1 and 2 for long periods (> 24 hours) without transiting to state 3, and it even showed a pause of almost 3 days without deep dives, see Fig 9 for dives 1345-1894. This indicates that feeding occurs infrequently and that narwhals at least during summer and fall may have extended periods without feeding activity (see also [6]). However, the median of these pauses without state 3 dives was 44 minutes and the mean was 2 hours.

## Discussion

In this study, we investigate different multivariate HMMs with covariate effects for modelling the diving activity of a narwhal in the vertical dimension in the water column. Although narwhals show relatively little behavioural plasticity [6, 7, 16], the present analysis is based on a sample of only one individual and there is therefore obvious limits to how far reaching conclusions that can be drawn from the diving behaviour of this individual. However, the value in the present analysis is the extraordinarily long data set and it is therefore also useful for examining the application of HMM methods as a tool for analyzing ontogenetic diving activity. The value of the sample includes the option for describing diurnal patterns in diving behaviour, during the fall migration.

We extend the existing HMMs for diving behaviour of marine mammals to allow for dependence between state distributions, and show that the dependence has some impact on the conclusions drawn about the diving behaviour. We find that statistically the correlated model outperforms the independent model, that the log-normal model outperforms the gamma model, and more importantly, conclusions on the diving behaviour differ between the models. The main differences are that the correlated models estimate more variable state distributions of MD and DT compared to the uncorrelated models. Thus, a major biological insight from the analysis of the correlated model is that variability is larger in behavioural states 1 and 2, but not in state 3. In the dependent log-normal model 56.3% of the dives are for feeding, compared to 60.5% in the independent log-normal model, under the assumption that states 2 and 3 in fact are representing feeding states in both models. Even if it is only a proportion of the dives that are not for feeding, it can be assumed that it is approximately the same proportion for the correlated and the independent models, and it is still a relatively large proportion of the diving effort that is allocated to feeding activities. This provides an important ecological insight that is useful when comparing feeding activities for whales inhabiting different ocean parts with different prey availability. Finally, ignoring the dependence between response variables leads to wrongly estimated standard deviations on parameter estimates, and thus confidence intervals are no longer valid.

The correlations between the post-dive duration and diving depth and duration are found to be vanishing. However, the post-dive response variable probably covers different behaviours that can not be distinguished from this data, such as recovering from a deep dive, resting between bouts of dives, social activities, travelling, etc.

Direct observations of feeding events were limited to the first week of the diving data but the depths where feeding events were detected served as a valid proxy for the depth threshold between behavioural state 2 and state 3. The observation that feeding events involve deep dives (≥ 350*m*) is also supported by studies of the buzzing activity during dives to different depths for narwhals travelling in the same area and time of the year as the whale included in this study [7].

Transition from state 1 to presumed feeding activity is more likely to be to state 3 with deep dives, and rarely goes to state 2 from state 1. Diving activity in state 3 usually last for a series of dives (5-10) perhaps indicating that specific layers of prey is being detected and explored for a series of dives before the whale needs to spend an extended period at the surface. The post dive time is typically around 6.9 minutes after a state 3 dive, whereas it is typically only 2.6 minutes after a state 2 dive. The whale probably needs to spend more time at the surface to recover from nitrogen tissue tension following a longer breath-hold diving activity. Williams et al. (2011) [25] calculated that the oxygen stores in tissues from narwhals of similar size as the one in this study would support dives of less than 20 min and that energy saving during gliding on descent might increase this calculated aerobic dive limit to up to 24 min. The deep dives in state 3 in this study seem to be in good agreement with these physiological limitations.

Even though detailed dive information supplemented by data on feeding events have been available for this analysis it may still not be adequate for describing the important drivers of diving behaviour. Both physiological constrains and reproductive state as well as environmental conditions may influence the diving activity to an extent that cannot be fully discerned in HMM analysis of dive series. For logistical reasons it is very difficult if not impossible to obtain information on all factors that affect the diving behaviour. However, the analysis of dive series provides a minimal insight into the integrated effect of the various factors driving the diving behaviour and the major advantage of the HMM analysis probably relies in the objective inter- and intra-specific comparison of diving activity. This study demonstrated the usefulness of HMMs for gaining insight to the hidden structures of dive patterns, something that is difficult to achieve with traditional statistics. It will be important to apply HMM techniques to larger data sets of diving activity from several whales to estimate how effective HMMs are for providing broader ecological insight to energetics and multispecies effects of whale predation.

## Supporting information

S1 Table Different models for covariate effects on the transition probabilities between behavioural states. The predictors *η*_*ij*_ relate to the transition probabilities.
(PDF)

S2 Table Estimates of the model parameters of the state distributions and their 95% confidence intervals in model 1 for correlated log-normal distribution. In state *i*, *μ*_*i*_ and *σ*_*i*_ are the log-mean and log-standard deviation of the correlated log-normal distribution. Index MD stands for Maximum Depth, DT stands for Dive Duration and PD stands for Post-Dive time. The depth is measured in meters, and time in seconds. The confidence intervals were computed from the Hessian of the negative log-likelihood function, i.e., based on the inverse of the observed Fisher information.
(PDF)

S3 TableEstimates of the model parameters of covariate effects and their 95% confidence intervals in model 1 for correlated log-normal distribution.The spline effects of hour are denoted by $H_{ij}^{t}=\sum_{k}\delta_{ij}^{\left(k\right)}h_{k}^{t}$, of *τ*_*t*_ by $T_{ij}^{t}=\sum_{k}\theta_{ij}^{\left(k\right)}s_{k}^{t}$, and of *d*_*t*_ by $D_{ij}^{t}=\sum_{k}\zeta_{ij}^{\left(k\right)}d_{k}^{t}$ for *k* = 1, 2, 3 and *i*, *j* = 1, 2, 3; *i* ≠ *j*.(PDF)

S1 Data Data analyzed in the paper. Data columns are: DiveNumber: Number of dive; Date: Date of dive; StartTime: Start time in hh:mm:ss of dive; MaxDepth: Maximum depth reach in dive in meters; Duration: Duration of dive in minutes; PostDiveDur: Duration of time spent in the surface (above 20 m) after the dive in minutes.
(ZIP)
