# Correction: Understanding narwhal diving behaviour using Hidden Markov Models with dependent state distributions and long range dependence

**DOI:** 10.1371/journal.pcbi.1010428

**Published:** 2022-08-05

**Authors:** Manh Cuong Ngô, Mads Peter Heide-Jørgensen, Susanne Ditlevsen

The Data Availability statement for this paper is incorrect. The correct statement is: The authors confirm that all data underlying the findings are fully available without restriction. The source code and original data are available here: https://gitlab.com/whales-reseach-code/hmm_paper

The processed data is available in the Supporting Information.
