# Supplementary material for: Understanding narwhal diving behaviour using Hidden Markov Models with dependent state distributions and long range dependence
Source: PLoS Comput Biol. 2019 Mar 14;15(3):e1006425. doi: 10.1371/journal.pcbi.1006425 (PMC6417660; doi:10.1371/journal.pcbi.1006425)
Supplement: S1 Table — The predictors ηij relate to the transition probabilities. (PDF) [file pcbi.1006425.s001.pdf]

Table S1. Different models for covariate effects on the transition probabilities between behavioural states. The predictors η_{ij} relate to the transition probabilities.

Model	$\eta_{12}^{(t)}$	$\eta_{13}^{(t)}$	$\eta_{21}^{(t)}$	$\eta_{23}^{(t)}$	$\eta_{31}^{(t)}$	$\eta_{32}^{(t)}$
1	$\alpha_{00}+\alpha_{10}\tau_t+\alpha_{20}\tau_t^2$ $+\sum_i \delta_{i0}h_i$	$\alpha_{01}+\alpha_{11}\tau_t+\alpha_{21}\tau_t^2$ $+\sum_i \delta_{i1}h_i$	$\beta_{00}+\beta_{10}\tau_t+\beta_{20}\tau_t^2$ $+\sum_i \theta_{i0}h_i$	$\beta_{01}+\beta_{11}\tau_t+\beta_{21}\tau_t^2$ $+\sum_i \theta_{i1}h_i$	$\gamma_{00}+\gamma_{10}d_t+\gamma_{20}d_t^2$ $+\sum_i \zeta_{i0}h_i$	$\gamma_{01}+\gamma_{11}d_t+\gamma_{21}d_t^2$ $+\sum_i \zeta_{i1}h_i$
2	$\alpha_{00}+\sum_i \delta_{i0}h_i$	$\alpha_{01}+\sum_i \delta_{i1}h_i$	$\beta_{00}+\sum_i \theta_{i0}h_i$	$\beta_{01}+\sum_i \theta_{i1}h_i$	$\gamma_{00}+\sum_i \zeta_{i0}h_i$	$\gamma_{01}+\sum_i \zeta_{i1}h_i$
3	$\alpha_{00}+\alpha_{10}\tau_t+\alpha_{20}\tau_t^2$	$\alpha_{01}+\alpha_{11}\tau_t+\alpha_{21}\tau_t^2$	$\beta_{00}+\beta_{10}\tau_t+\beta_{20}\tau_t^2$	$\beta_{01}+\beta_{11}\tau_t+\beta_{21}\tau_t^2$	$\gamma_{00}+\gamma_{10}d_t+\gamma_{20}d_t^2+$ $\sum_i \zeta_{i0}h_i$	$\gamma_{01}+\gamma_{11}d_t+\gamma_{21}d_t^2+$ $\sum_i \zeta_{i1}h_i$
4	$\alpha_{00}+\alpha_{10}\tau_t+\alpha_{20}\tau_t^2$	$\alpha_{01}+\alpha_{11}\tau_t+\alpha_{21}\tau_t^2$	$\beta_{00}+\beta_{10}\tau_t+\beta_{20}\tau_t^2$	$\beta_{01}+\beta_{11}\tau_t+\beta_{21}\tau_t^2$	$\gamma_{00}+\gamma_{10}d_t+\gamma_{20}d_t^2$	$\gamma_{01}+\gamma_{11}d_t+\gamma_{21}d_t^2$
5	α_{00}	α_{01}	β_{00}	β_{01}	γ_{00}	γ_{01}
6	$\alpha_{00}+\alpha_{10}\tau_t$	$\alpha_{01}+\alpha_{11}\tau_t$	$\beta_{00}+\beta_{10}\tau_t$	$\beta_{01}+\beta_{11}\tau_t$	$\gamma_{00}+\gamma_{10}b_t$	$\gamma_{01}+\gamma_{11}b_t$
7	$\alpha_{00}+\alpha_{10}\tau_t$	$\alpha_{01}+\alpha_{11}\tau_t$	$\beta_{00}+\beta_{10}\tau_t$	$\beta_{01}+\beta_{11}\tau_t$	$\gamma_{00}+\gamma_{10}d_t$	$\gamma_{01}+\gamma_{11}d_t$
8	$\alpha_{00}+\alpha_{10}\tau_t+\alpha_{20}\tau_t^2$	$\alpha_{01}+\alpha_{11}\tau_t+\alpha_{21}\tau_t^2$	β_{00}	$\beta_{01}+\beta_{11}\tau_t+\beta_{21}\tau_t^2$	$\gamma_{00}+\gamma_{10}d_t+\gamma_{20}d_t^2$	$\gamma_{01}+\gamma_{11}d_t+\gamma_{21}d_t^2$
9	α_{00}	$\alpha_{01}+\alpha_{11}\tau_t+\alpha_{21}\tau_t^2$	β_{00}	$\beta_{01}+\beta_{11}\tau_t+\beta_{21}\tau_t^2$	$\gamma_{00}+\gamma_{10}d_t+\gamma_{20}d_t^2$	$\gamma_{01}+\gamma_{11}d_t+\gamma_{21}d_t^2$
10	$\alpha_{00}+\alpha_{10}\tau_t$	$\alpha_{01}+\alpha_{11}\tau_t+\alpha_{21}\tau_t^2$	$\beta_{00}+\beta_{10}\tau_t$	$\beta_{01}+\beta_{11}\tau_t+\beta_{21}\tau_t^2$	$\gamma_{00}+\gamma_{10}d_t+\gamma_{20}d_t^2$	$\gamma_{01}+\gamma_{11}d_t+\gamma_{21}d_t^2$
11	$\alpha_{10}\tau_t+\alpha_{20}\tau_t^2$ $+\sum_i \delta_{i0}h_i$	$\alpha_{11}\tau_t+\alpha_{21}\tau_t^2$ $+\sum_i \delta_{i1}h_i$	$\beta_{10}\tau_t+\beta_{20}\tau_t^2$ $+\sum_i \theta_{i0}h_i$	$\beta_{11}\tau_t+\beta_{21}\tau_t^2$ $+\sum_i \theta_{i1}h_i$	$\gamma_{10}d_t+\gamma_{20}d_t^2$ $+\sum_i \zeta_{i0}h_i$	$\gamma_{11}d_t+\gamma_{21}d_t^2$ $+\sum_i \zeta_{i1}h_i$
12	$\alpha_{00}+\alpha_{10}\tau_t+\alpha_{20}\tau_t^2$	$\alpha_{01}+\alpha_{11}\tau_t+\alpha_{21}\tau_t^2$ $+\sum_i \delta_{i1}h_i$	$\beta_{00}+\beta_{10}\tau_t+\beta_{20}\tau_t^2$	$\beta_{01}+\beta_{11}\tau_t+\beta_{21}\tau_t^2$ $+\sum_i \theta_{i1}h_i$	$\gamma_{00}+\gamma_{10}d_t+\gamma_{20}d_t^2$ $+\sum_i \zeta_{i0}h_i$	$\gamma_{01}+\gamma_{11}d_t+\gamma_{21}d_t^2$ $+\sum_i \zeta_{i1}h_i$
13	α_{00}	$\alpha_{01}+\alpha_{11}\tau_t+\alpha_{21}\tau_t^2$	β_{00}	$\beta_{01}+\beta_{11}\tau_t+\beta_{21}\tau_t^2$	$\gamma_{00}+\gamma_{10}d_t+\gamma_{20}d_t^2$ $+\sum_i \zeta_{i0}h_i$	$\gamma_{01}+\gamma_{11}d_t+\gamma_{21}d_t^2$ $+\sum_i \zeta_{i0}h_i$
14	$\alpha_{00}+\sum_i \delta_{i0}h_i$	$\alpha_{01}+\alpha_{11}\tau_t+\alpha_{21}\tau_t^2$ $+\sum_i \delta_{i1}h_i$	$\beta_{00}+\sum_i \theta_{i0}h_i$	$\beta_{01}+\beta_{11}\tau_t+\beta_{21}\tau_t^2$ $\sum_i \theta_{i1}h_i$	$\gamma_{00}+\gamma_{10}d_t+\gamma_{20}d_t^2$	$\gamma_{01}+\gamma_{11}d_t+\gamma_{21}d_t^2$ $+\sum_i \zeta_{i1}h_i$
