# Supplementary material for: Understanding narwhal diving behaviour using Hidden Markov Models with dependent state distributions and long range dependence
Source: PLoS Comput Biol. 2019 Mar 14;15(3):e1006425. doi: 10.1371/journal.pcbi.1006425 (PMC6417660; doi:10.1371/journal.pcbi.1006425)
Supplement: S2 Table — In state i, μi and σi are the log-mean and log-standard deviation of the correlated log-normal distribution. Index MD stands for Maximum Depth, DT stands for Dive Duration and PD stands for Post-Dive time. The depth is measured in meters, and time in seconds. The confidence intervals were computed from the Hessian of the negative log-likelihood function, i.e., based on the inverse of the observed Fisher information. (PDF) [file pcbi.1006425.s002.pdf]

Table S2. Estimates of the model parameters of the state distributions and their 95% confidence intervals in model 1 for correlated Log-normal distribution. In state i, μ_i and σ_i are the log-mean and log-standard deviation of the correlated log-normal distribution. Index MD stands for Maximum Depth, DT stands for Dive Duration and PD stands for Post-Dive time. The depth is measured in meters, and time in seconds. The confidence intervals were computed from the Hessian of the negative log-likelihood function, i.e., based on the inverse of the observed Fisher information.

Correlated log-normal distribution		
	Estimate	95% CI
μ_1^{MD}	2.61	$[2.56, 2.66]$
μ_2^{MD}	4.78	$[4.73, 4.84]$
μ_3^{MD}	6.11	$[6.11, 6.12]$
σ_1^{MD}	1.36	$[1.33, 1.39]$
σ_2^{MD}	0.77	$[0.72, 0.81]$
σ_3^{MD}	0.18	$[0.17, 0.19]$
μ_1^{DT}	1.50	$[1.48, 1.52]$
μ_2^{DT}	1.80	$[1.77, 1.82]$
μ_3^{DT}	2.46	$[2.45, 2.46]$
σ_1^{DT}	0.50	$[0.49, 0.51]$
σ_2^{DT}	0.43	$[0.41, 0.46]$
σ_3^{DT}	0.14	$[0.14, 0.14]$
μ_1^{PD}	1.26	$[1.22, 1.3]$
μ_2^{PD}	0.86	$[0.83, 0.88]$
μ_3^{PD}	1.73	$[1.71, 1.75]$
σ_1^{PD}	1.13	$[1.10, 1.15]$
σ_2^{PD}	0.43	$[0.41, 0.45]$
σ_3^{PD}	0.53	$[0.52, 0.55]$
ρ_1	0.56	$[0.53, 0.58]$
ρ_2	0.81	$[0.78, 0.83]$
ρ_3	0.46	$[0.43, 0.50]$
