# Supplementary material for: Understanding narwhal diving behaviour using Hidden Markov Models with dependent state distributions and long range dependence
Source: PLoS Comput Biol. 2019 Mar 14;15(3):e1006425. doi: 10.1371/journal.pcbi.1006425 (PMC6417660; doi:10.1371/journal.pcbi.1006425)
Supplement: S3 Table — The spline effects of hour are denoted by Hijt=∑kδij(k)hkt, of τt by Tijt=∑kθij(k)skt, and of dt by Dijt=∑kζij(k)dkt for k = 1, 2, 3 and i, j = 1, 2, 3; i ≠ j. (PDF) [file pcbi.1006425.s003.pdf]

Table S3. Estimates of the model parameters of covariate effects and their 95% confidence intervals in model 1 for correlated Log-normal distribution. The spline effects of hour are denoted by $H_{ij}^t = \sum_k \delta_{ij}^{(k)} h_k^t$, of τ_t by $T_{ij}^t = \sum_k \theta_{ij}^{(k)} s_k^t$, and of d_t by $D_{ij}^t = \sum_k \zeta_{ij}^{(k)} d_k^t$ for $k = 1, 2, 3$ and $i, j = 1, 2, 3$; $i \neq j$.

Correlated log-normal distribution		
	Estimate	95% CI
α_{00}	-3.82	$[-5.30, -2.34]$
α_{01}	-0.97	$[-1.57, -0.37]$
β_{00}	0.08	$[-1.18, 1.33]$
β_{01}	-1.74	$[-3.22, -0.263]$
γ_{00}	1.88	$[1.10, 2.66]$
γ_{01}	-3.08	$[-5.15, -1.02]$
$\theta_{12}^{(1)}$	1.95	$[0.11, 3.80]$
$\theta_{12}^{(2)}$	-1.71	$[-3.77, 0.36]$
$\theta_{12}^{(3)}$	-2.48	$[-5.48, 0.52]$
$\theta_{13}^{(1)}$	-3.11	$[-4.39, -1.82]$
$\theta_{13}^{(2)}$	-6.60	$[-8.28, -4.92]$
$\theta_{13}^{(3)}$	-7.08	$[-10.5, -3.61]$
$\theta_{21}^{(1)}$	0.19	$[-1.58, 1.97]$
$\theta_{21}^{(2)}$	-0.57	$[-2.39, 1.25]$
$\theta_{21}^{(3)}$	-0.51	$[-4.10, 3.08]$
$\theta_{23}^{(1)}$	-5.04	$[-8.82, -1.26]$
$\theta_{23}^{(2)}$	-5.39	$[-7.13, -3.66]$
$\theta_{23}^{(3)}$	-0.48	$[-1.93, 0.98]$
$\zeta_{31}^{(1)}$	-2.83	$[-3.75, -1.92]$
$\zeta_{31}^{(2)}$	-5.35	$[-6.61, -4.1]$
$\zeta_{31}^{(3)}$	-0.84	$[-2.51, 0.83]$
$\zeta_{32}^{(1)}$	-4.83	$[-6.38, -3.28]$
$\zeta_{32}^{(2)}$	-8.80	$[-10.30, -7.28]$
$\zeta_{32}^{(3)}$	-0.38	$[-2.76, 2.00]$
$\delta_{12}^{(1)}$	0.41	$[-1.78, 2.60]$
$\delta_{12}^{(2)}$	2.53	$[1.33, 3.73]$
$\delta_{12}^{(3)}$	-0.06	$[-2.34, 2.22]$
$\delta_{13}^{(1)}$	0.21	$[-0.82, 1.23]$
$\delta_{13}^{(2)}$	0.08	$[-0.42, 0.58]$
$\delta_{13}^{(3)}$	0.02	$[-1.06, 1.10]$
$\delta_{21}^{(1)}$	-2.77	$[-4.88, -0.66]$
$\delta_{21}^{(2)}$	-2.29	$[-3.34, -1.25]$
$\delta_{21}^{(3)}$	-3.46	$[-5.37, -1.55]$
$\delta_{23}^{(1)}$	-1.24	$[-3.48, 1.01]$
$\delta_{23}^{(2)}$	0.63	$[-0.93, 2.19]$
$\delta_{23}^{(3)}$	1.54	$[-0.37, 3.45]$
$\delta_{31}^{(1)}$	-2.32	$[-3.41, -1.23]$
$\delta_{31}^{(2)}$	-0.60	$[-1.14, -0.06]$
$\delta_{31}^{(3)}$	-2.49	$[-3.59, -1.39]$
$\delta_{32}^{(1)}$	4.45	$[0.99, 7.90]$
$\delta_{32}^{(2)}$	6.28	$[4.05, 8.51]$
$\delta_{32}^{(3)}$	4.94	$[1.47, 8.41]$
